# Supplementary material for: Ga2Te3-Based Composite Anodes for High-Performance Sodium-Ion Batteries
Source: Materials (Basel). 2022 Sep 8;15(18):6231. doi: 10.3390/ma15186231 (PMC9504644; doi:10.3390/ma15186231)
Supplement: Supplementary file 1 [file materials-15-06231-s001.zip › materials-1890047-supplementary.pdf]

SUPPORTING INFORMATION

Ga_2Te_3-Based Composite Anodes for High-Performance Sodium-Ion Batteries

Vo Pham Hoang Huy, Il Tae Kim * and Jaehyun Hur *

Department of Chemical and Biological Engineering, Gachon University, Seongnam 13120, Gyeonggi, Korea
* Correspondence: itkim@gachon.ac.kr (I.T.K.); jhhur@gachon.ac.kr (J.H.)

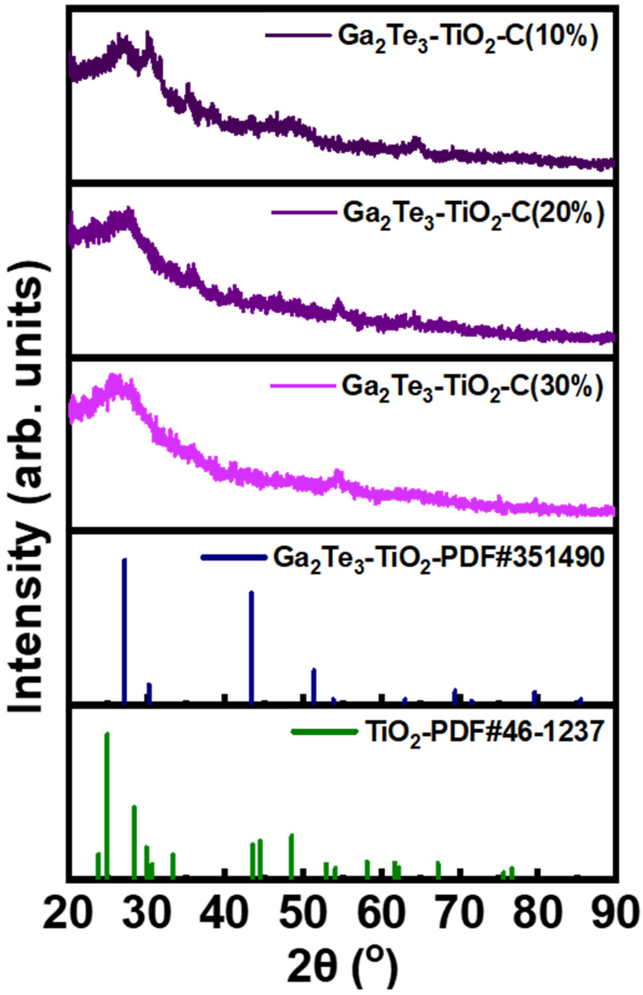

Figure S1. XRD pattern of Ga_2Te_3-TiO_2-C with different concentration of C.

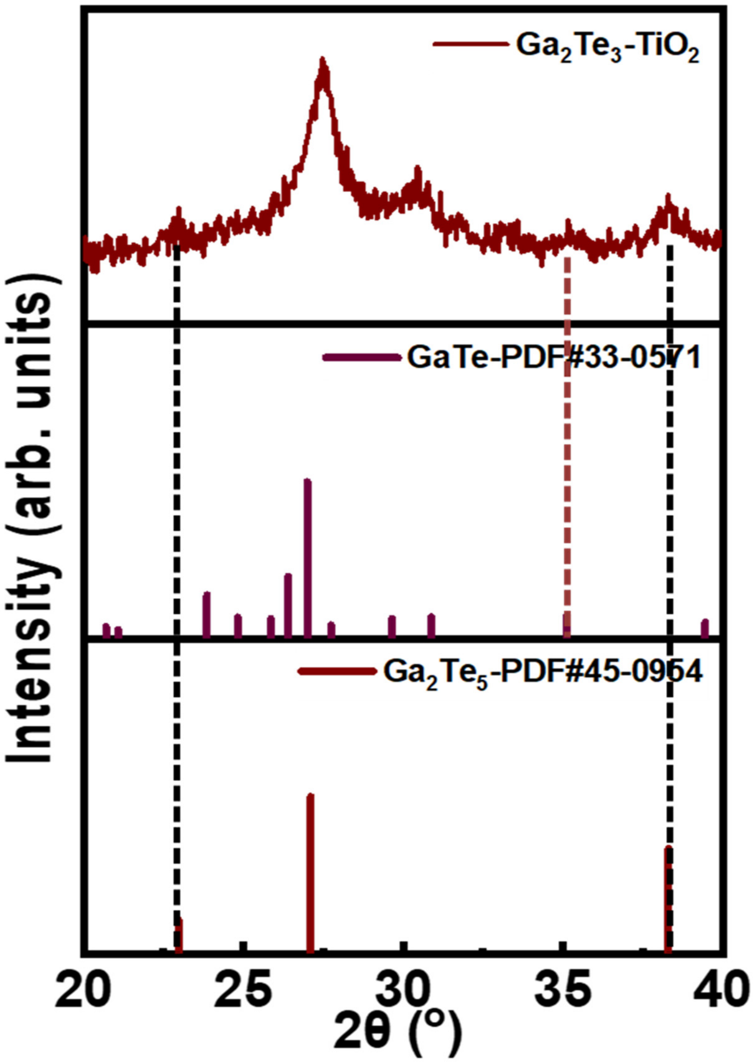

Figure S2. The existence of different forms of Ga_xTe_y (namely, Ga_2Te_5 and GaTe).

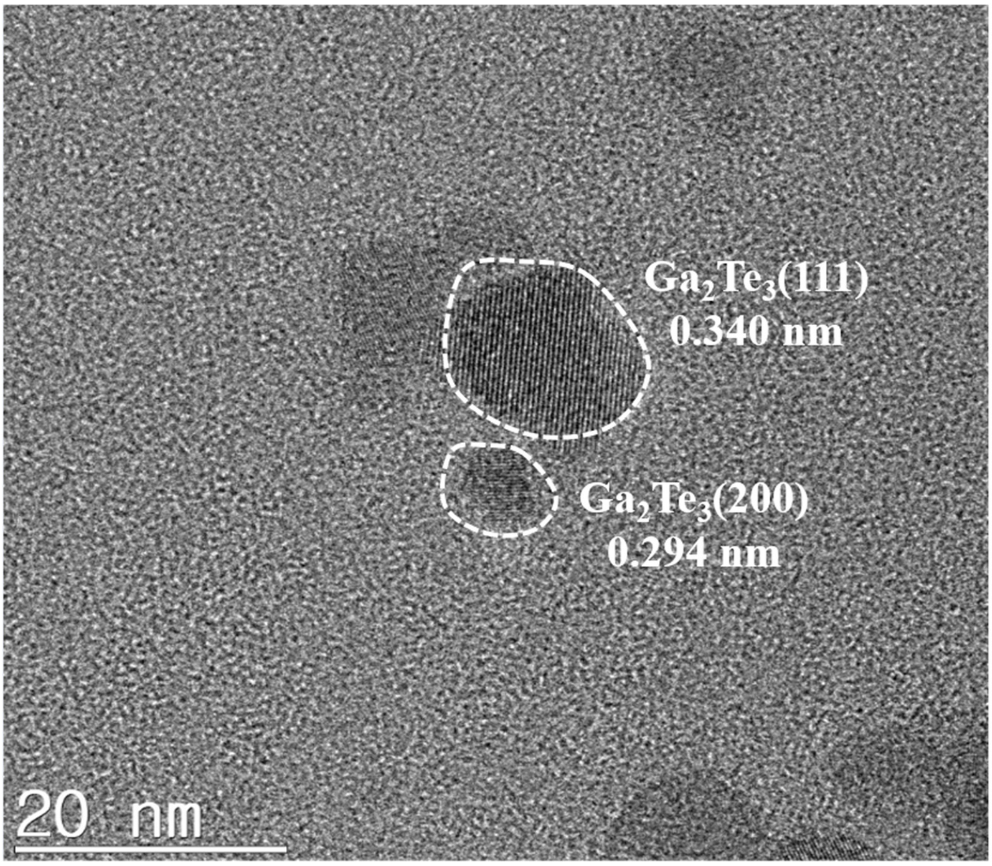

Figure S3. HRTEM image of Ga_2Te_3-TiO_2-C(10%).

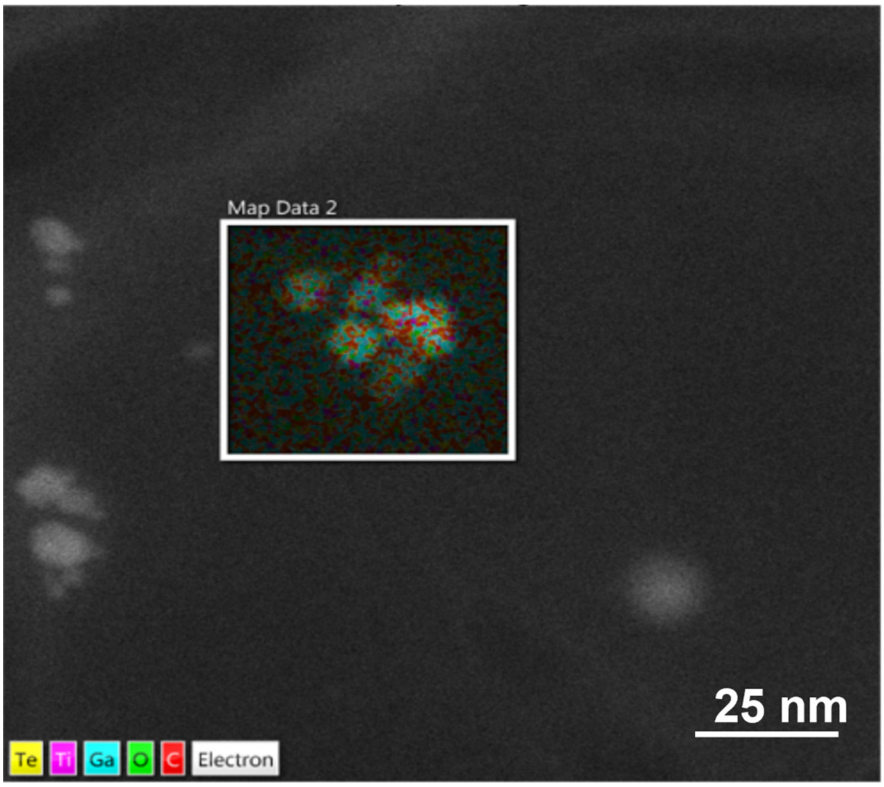

Figure S4. SEM image of distribution of the elements in Ga_2Te_3-TiO_2-C(10%).

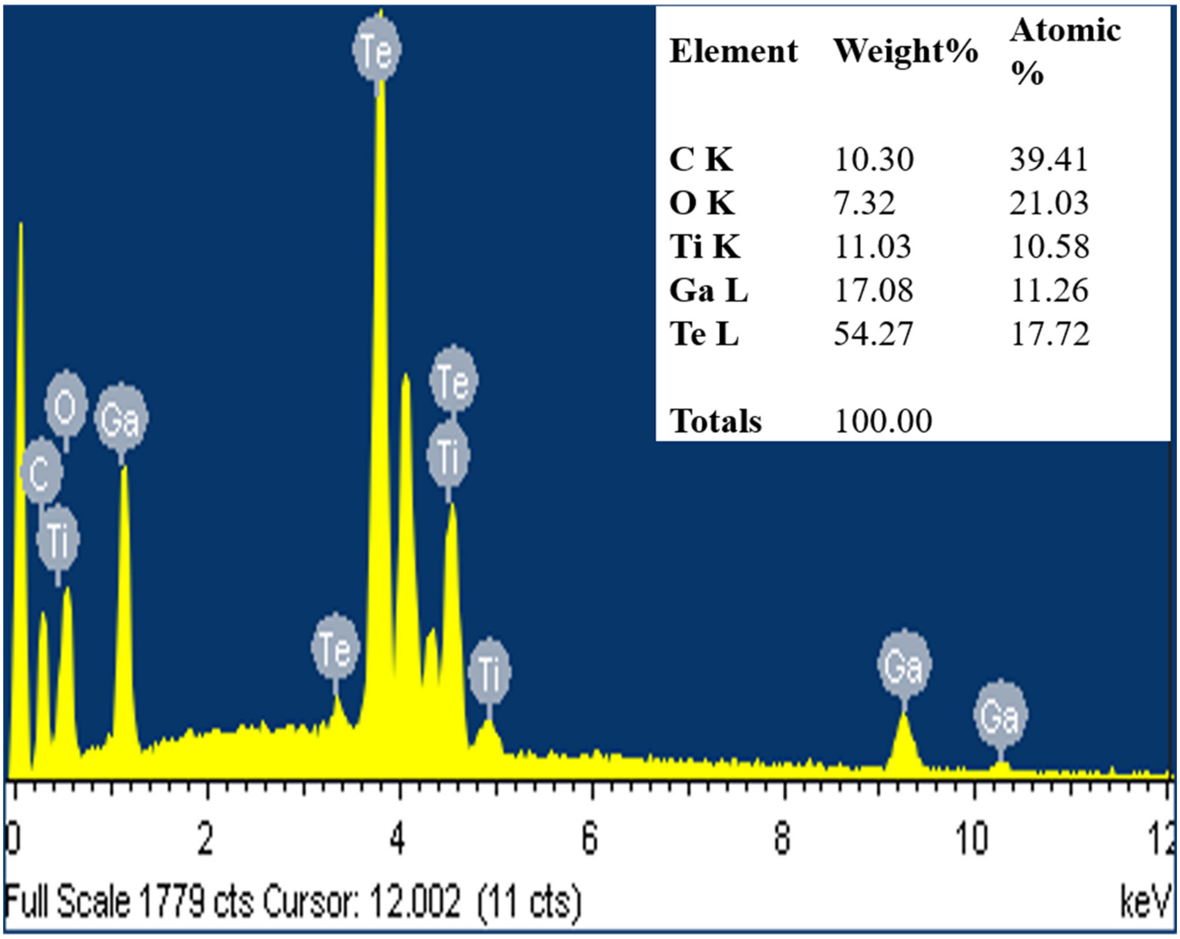

Figure S5. EDX spectrum of as-synthesized Ga_2Te_3-TiO_2-C(10%).

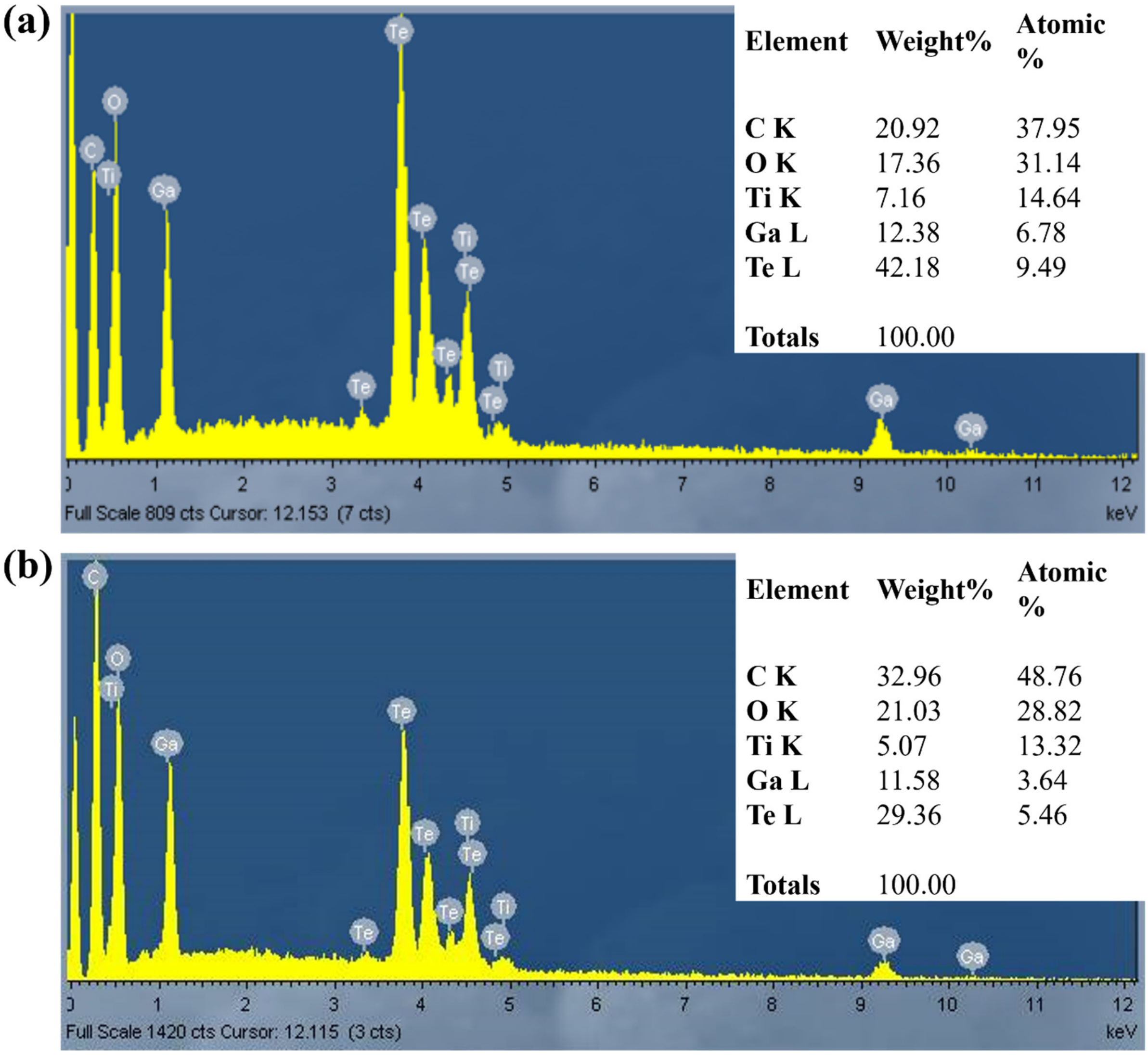

(a)

Element	Weight%	Atomic %
C K	20.92	37.95
O K	17.36	31.14
Ti K	7.16	14.64
Ga L	12.38	6.78
Te L	42.18	9.49
Totals	100.00	

(b)

Element	Weight%	Atomic %
C K	32.96	48.76
O K	21.03	28.82
Ti K	5.07	13.32
Ga L	11.58	3.64
Te L	29.36	5.46
Totals	100.00	

Figure S6. EDX analysis of (a) Ga_2Te_3-TiO_2-C(20%), and (b) Ga_2Te_3-TiO_2-C(30%).

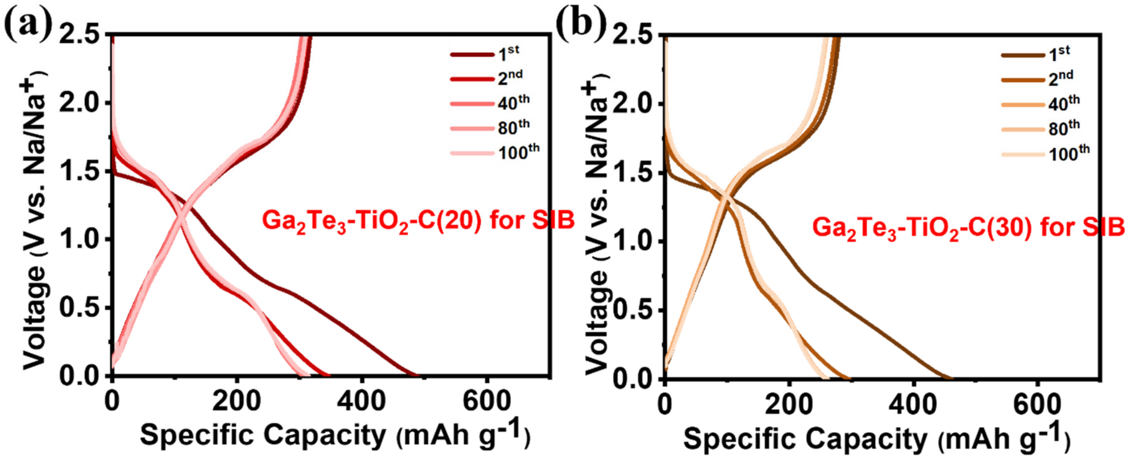

Figure S7. Galvanostatic discharge-charge profiles of (a) Ga_2Te_3-TiO_2-C(20%) and (b) Ga_2Te_3-TiO_2-C(30%) for SIBs.

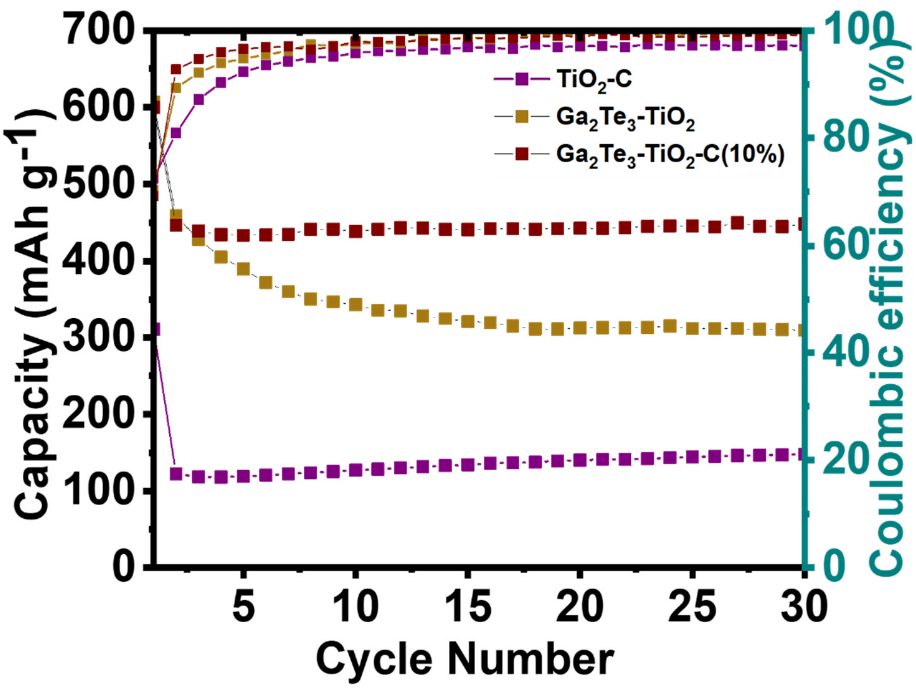

Figure S8. Cyclic performance of various electrodes at 100 mA g^{-1}.

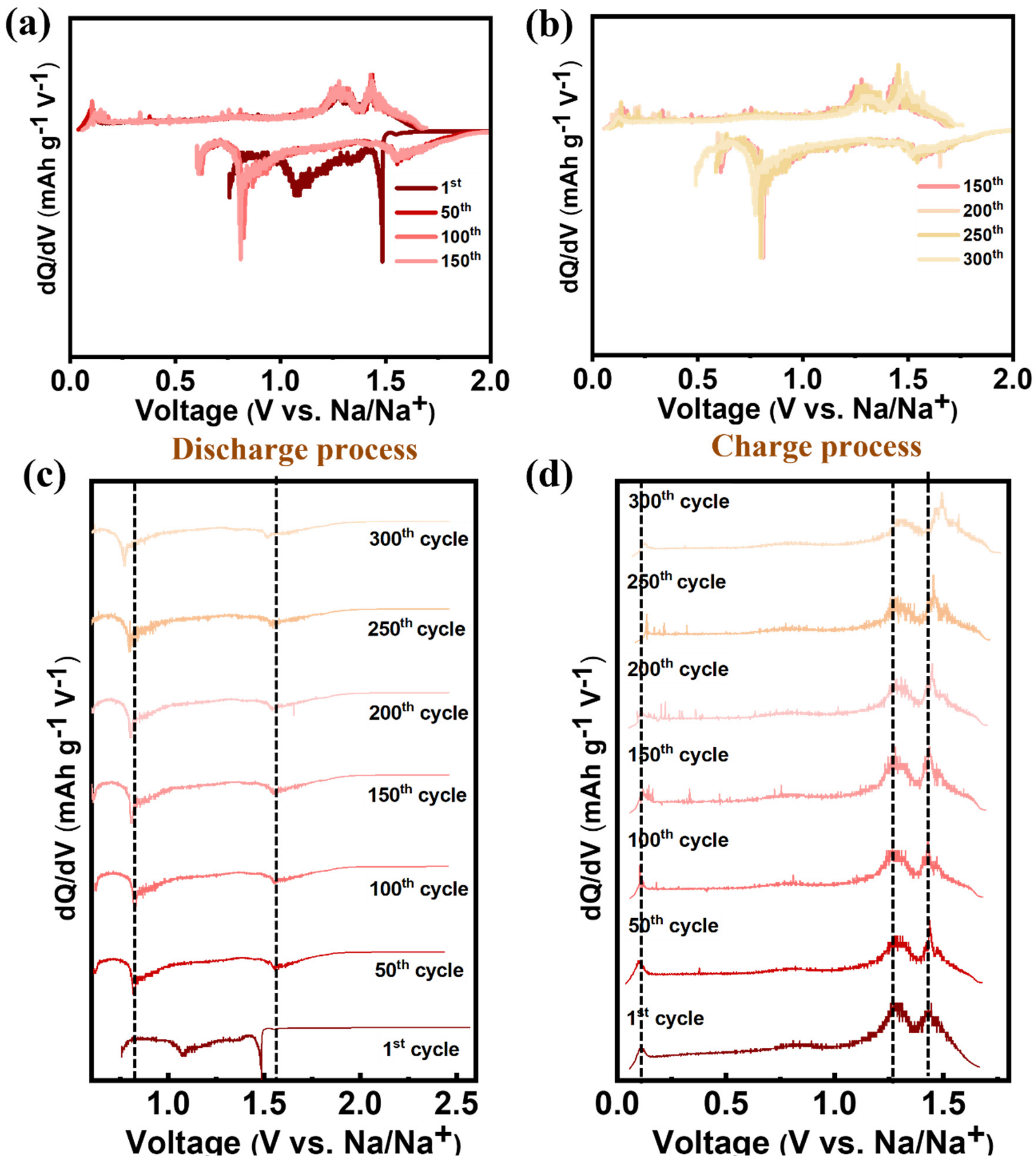

Figure S9. DCP profiles of Ga_2Te_3-TiO_2-C(10%) during 300 cycles measured at 100 mA g^{-1}: (a) 1−150 cycles, (b) 150−300 cycle. Enlarged view of (c) reduction and (d) oxidation peaks.

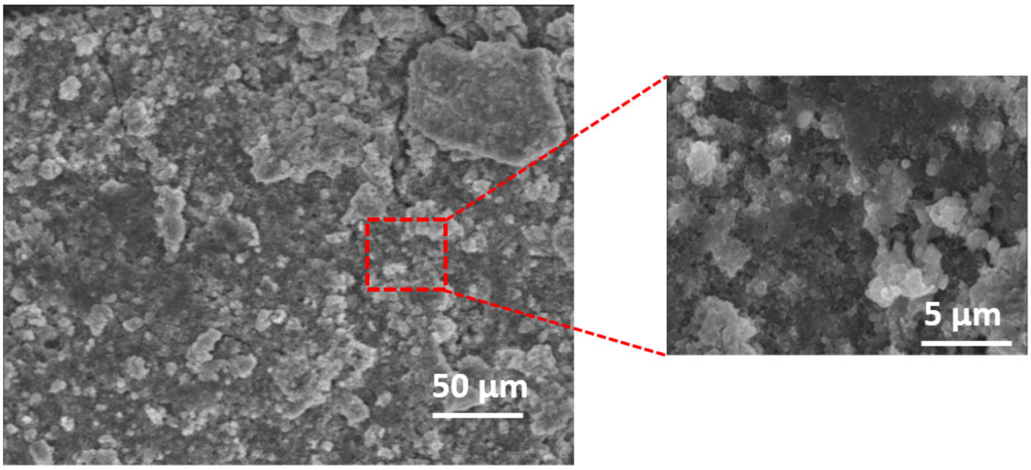

Figure S10. SEM image of Ga_2Te_3-TiO_2-C(10%) after 300 cycles.

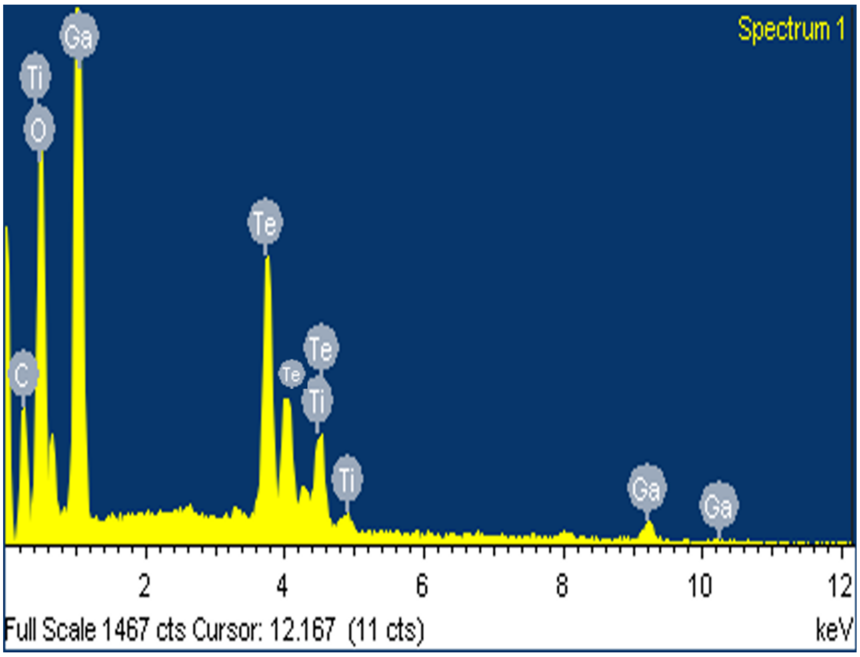

Figure S11. EDX analysis of Ga_2Te_3-TiO_2-C(10%) after 300 cycles.

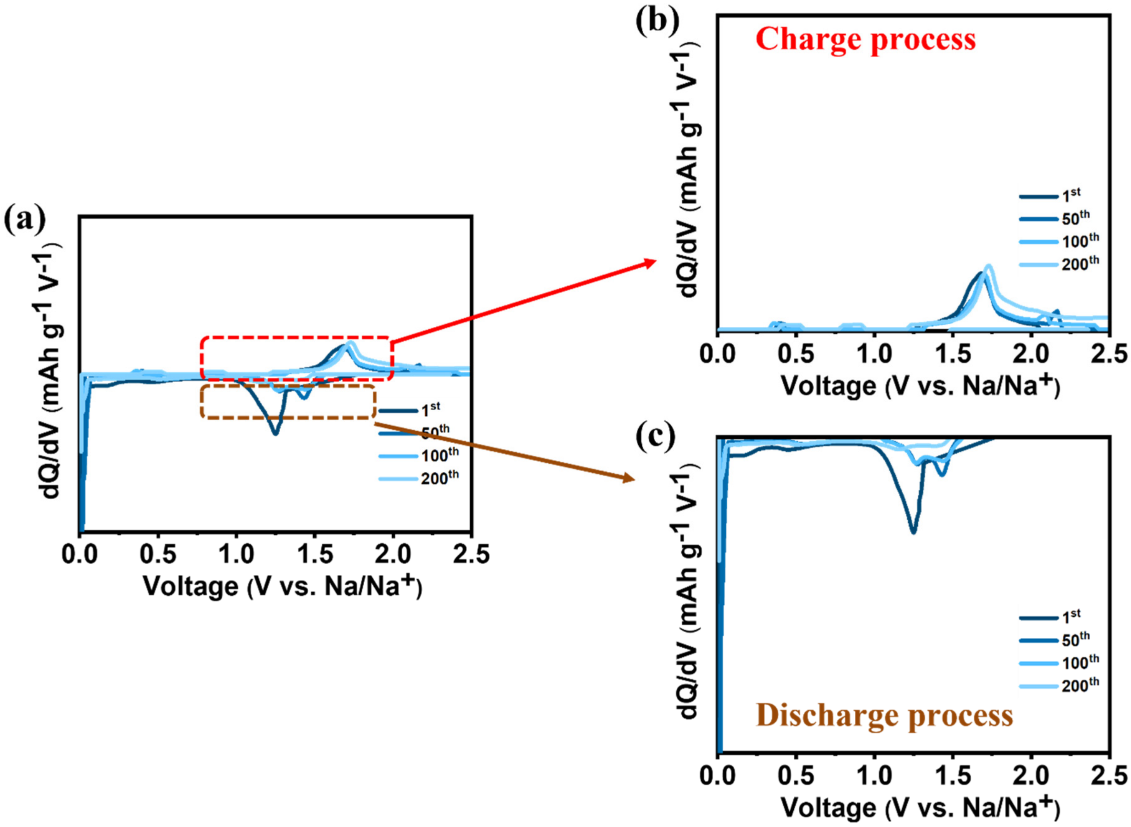

Figure S12. (a) DCP profiles of Ga_2Te_3-TiO_2-C(10%) during initial 200 cycles measured at 500 mA g^{-1}. Enlarged view of (b) oxidation and (c) reduction peaks.

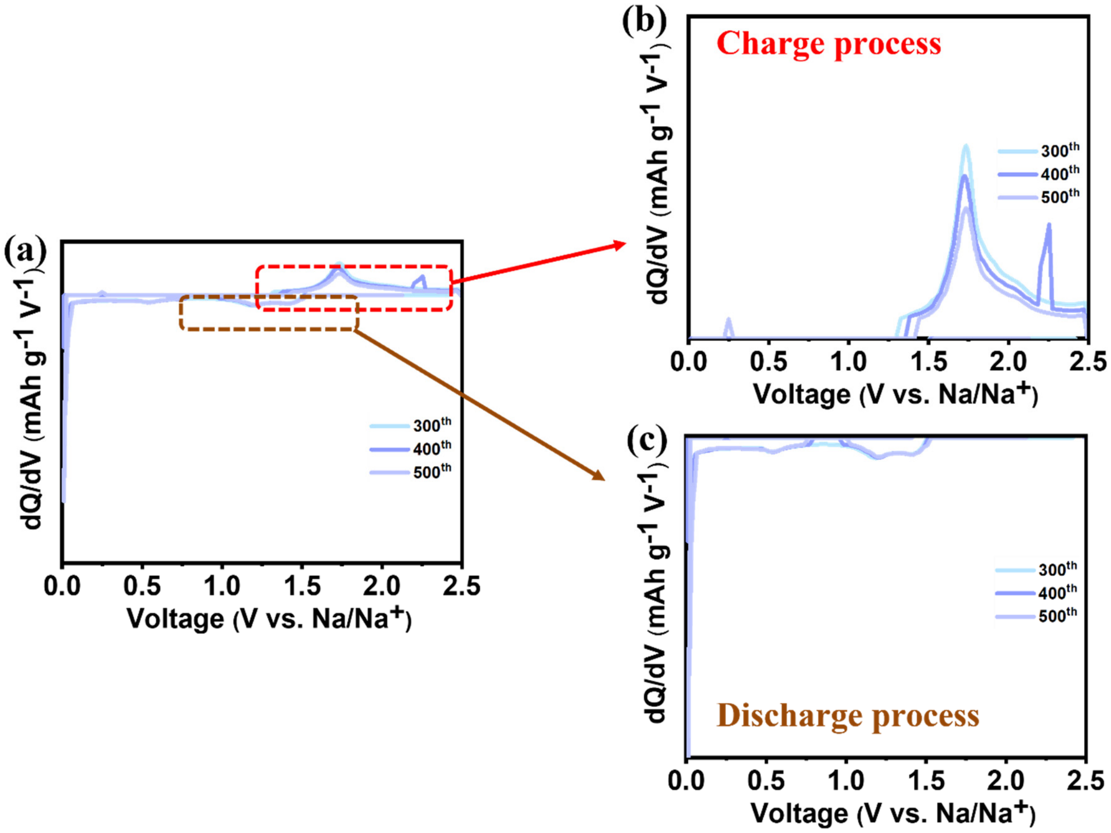

Figure S13. (a) DCP of Ga_2Te_3-TiO_2-C(10%) from 300 cycle to 500 cycles measured at 500 mA g^{-1}. Enlarged view of (b) oxidation and (c) reduction peaks.

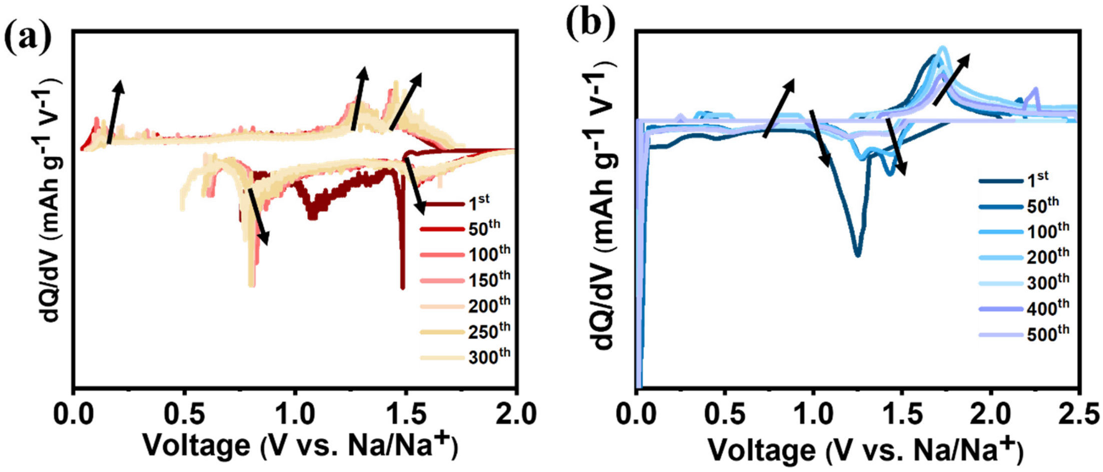

Figure S14. DCP profiles of Ga_2Te_3-TiO_2-C(10%) (a) at 100 mA g^{-1} during 300 cycles and (b) at 500 mA g^{-1} during 500 cycles.

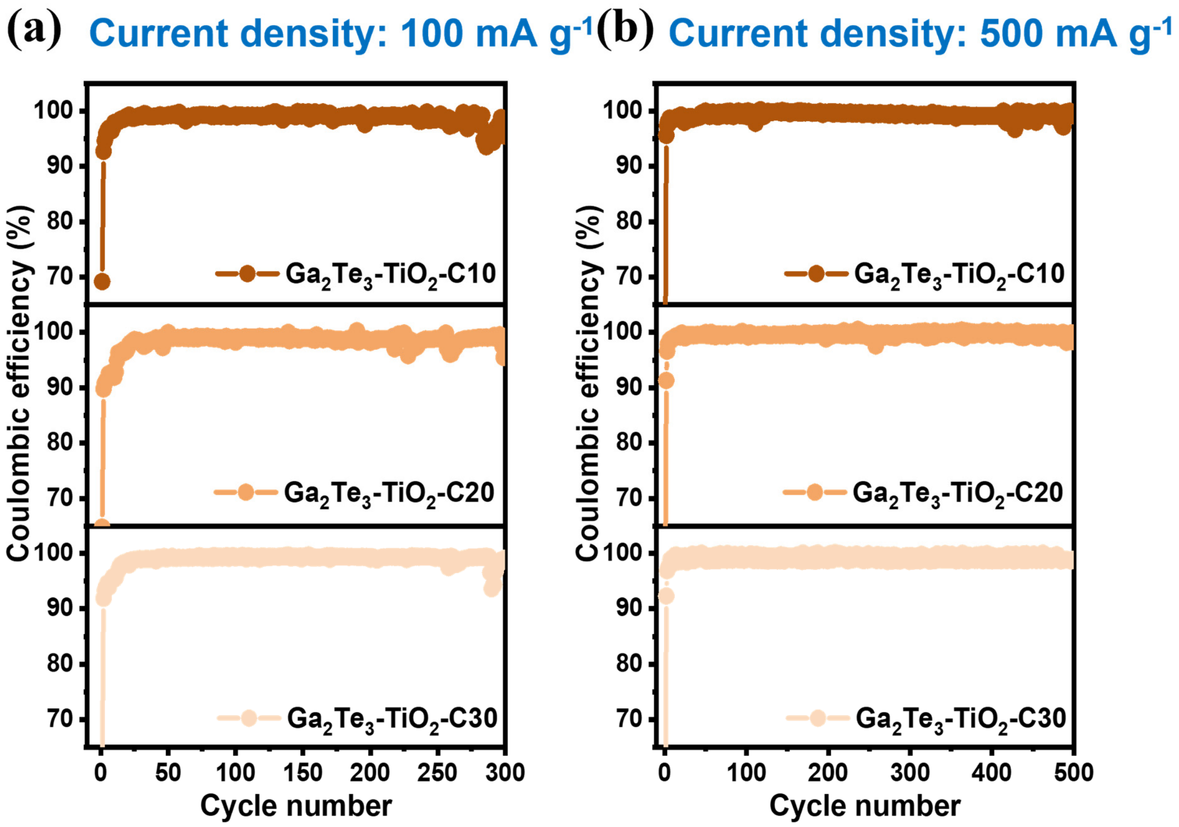

Figure S15. Coulombic efficieny of Ga_2Te_3-TiO_2 with different C content at current densities of (a) 100 and (b) 500 mA g^{-1}.

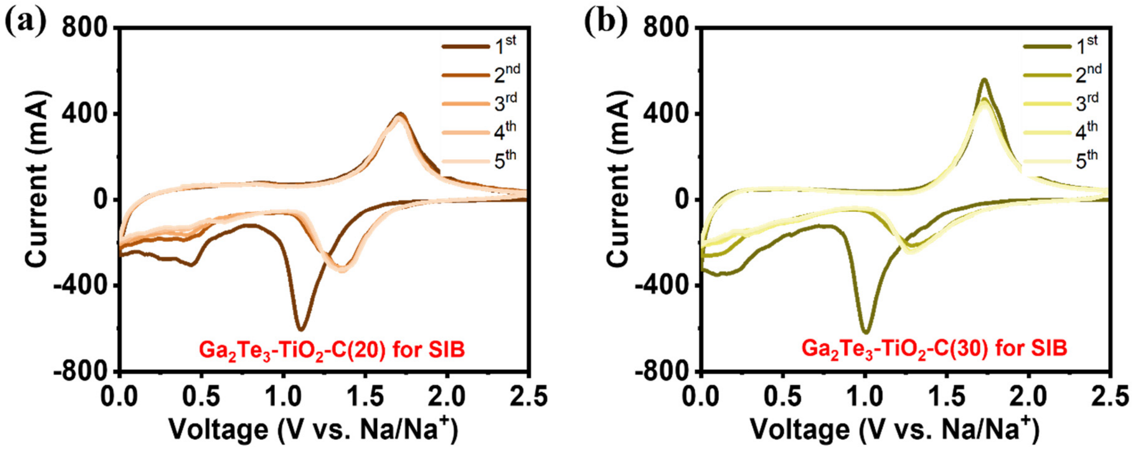

Figure S16. (a) CV curves of Ga_2Te_3-TiO_2C (20%), and (b) Ga_2Te_3-TiO_2-C (30%) for SIBs.

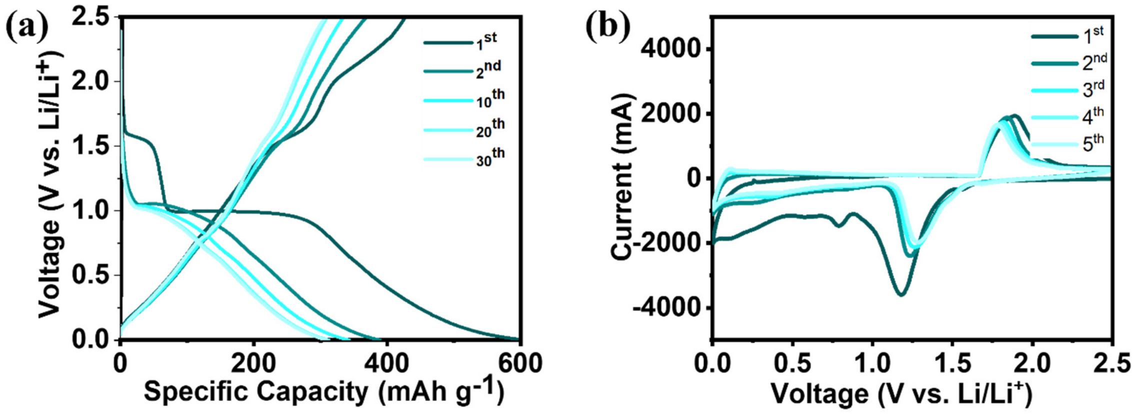

Figure S17. (a) Galvanostatic discharge-charge profiles of Ga_2Te_3-TiO_2 at a current density of 100 mA g^{-1}, (b) CV curves of Ga_2Te_3-TiO_2.

Table S1. Rate performance of Te-based composite anode for SIB

Anode	Rate capability	Capacity retention (%)	Ref.
Ga_2S_3-C	94 mAh g^{-1} at 2.0 A g^{-1}	78	[35]
Bi_2Te_3	339.40 mAh g^{-1} at 10.0 A g^{-1}	96	[36]
Sb_2Te_3-Te	226 mAh g^{-1} at 15 A g^{-1}	95	[37]
$NiTe_2$@N-doped carbon nanosheet	267 mAh g^{-1} at 10.0 A g^{-1}	95	[38]
$FeTe_2$@rGO	257 mAh g^{-1} at 3.0 A g^{-1}	93	39]
SbTe@C	413 mAh g^{-1} at 2.0 A g^{-1}	97	[40]
SnTe@C	117 mAh g^{-1} at 1.0 A g^{-1}	85	[41]
$MoTe_2$	185 mAh g^{-1} at 5.0 A g^{-1}	91	[42]
$MoTe_2$@3D porous carbon network	213 mAh g^{-1} at 20.0 A g^{-1}	56	[43]
$CoTe_2$@N-doped multichannel carbon fiber	152 mAh g^{-1} at 10 A g^{-1}	91	[44]

Table S2. Calculation of capacity contribution of Ga_2Te_3, TiO_2 and C in the Ga_2Te_3-TiO_2-C composite for SIB.

	**Ga_2Te_3**	**TiO_2**	**C**
The mole of Na-ion participating reaction	6.5	1	0.17
Molecular weight (g mol^{-1})	522.24	79.9	12
Calculated theoretical capacity (mAh g^{-1})	~333	~336	~380
Actual weight faction in the Ga_2Te_3-TiO_2-C composite	0.66	0.22	0.11
Contributed capacity (mAh g^{-1})	220	74	42
Capacity contribution (%)	65	22	13

Table S3. Calculation of theoretical capacity of Ga_2Te_3-TiO_2-C(10%) and Ga_2Te_3-TiO_2 for SIB

**Anode materials**	**Ga_2Te_3-TiO_2-C(10%)**			**Ga_2Te_3-TiO_2**		
Component	Ga_2Te_3	TiO_2	C	Ga_2Te_3	TiO_2	C
Theoretical weight faction	0.66	0.22	0.11	0.80	0.20	-
Theoretical capacity (mAh g^{-1})	~333	~336	~380	~333	~336	~380
Contributed theoretical capacity (mAh g^{-1})	~220	~74	~42	~266	~67	-
Total theoretical capacity (mAh g^{-1})		~336			~333	

Table S4. Coulombic efficiency variation of Ga_2Te_3-TiO_2-C (10%) at various cycle numbers measured at 100 mA g^{-1} for SIB

**Cycle number**	**Coulombic efficiency (%)**
1	69.18
10	97.92
20	98.82
50	99.30
100	99.30
150	99.82
200	98.69
250	98.84
300	98.49

Table S5. Coulombic efficiency variation of Ga_2Te_3-TiO_2-C (10%) at various cycle numbers measured at 500 mA g^{-1} for SIB

Cycle number	Coulombic efficiency (%)
1	59.18
10	98.64
20	99.29
50	99.99
100	99.05
200	99.69
300	99.30
400	99.12
500	99.59

Table. S6 Coulombic efficiency of Ga_2Te_3-TiO_2-C at current density of 100 mA g^{-1} during initial 10 cycles for SIB

**Cycle number**	**Coulombic efficiency (%)**		
	**Ga_2Te_3-TiO_2-C (10%)**	**Ga_2Te_3-TiO_2-C (20%)**	**Ga_2Te_3-TiO_2-C (30%)**
1st	69.18	64.80	60.47
2nd	92.74	89.79	91.86
3rd	94.68	90.82	93.29
4th	95.94	91.34	94.05
5th	96.54	91.69	94.62
6th	96.87	92.54	93.87
7th	96.95	92.44	94.66
8th	96.42	92.79	95.21
9th	96.97	91.93	95.72
10th	97.92	91.89	95.33

Table. S7 Coulombic efficiency of Ga_2Te_3-TiO_2-C at current density of 500 mA g^{-1} during initial 10 cycles for SIB

**Cycle number**	**Coulombic efficiency (%)**		
	**Ga_2Te_3-TiO_2-C (10%)**	**Ga_2Te_3-TiO_2-C (20%)**	**Ga_2Te_3-TiO_2-C (30%)**
1st	59.18	58.93	48.40
2nd	95.59	91.31	92.28
3rd	97.38	96.61	96.82
4th	98.09	97.86	97.18
5th	97.75	98.22	97.73
6th	98.71	98.56	97.92
7th	98.38	98.47	98.84
8th	98.19	98.85	97.92
9th	98.33	98.83	98.69
10th	98.64	97.92	98.22

Table. S8. Charge-transfer resistance (R_{ct}) of Ga_2Te_3-TiO_2-C for SIB

	**Ga_2Te_3-TiO_2-C (10%)**	**Ga_2Te_3-TiO_2-C (20%)**	**Ga_2Te_3-TiO_2-C (30%)**
1 cycle	100.4 Ω	180.72 Ω	130.7 Ω
5 cycles	125.6 Ω	148.8 Ω	183.3 Ω
20 cycles	99.41 Ω	124.4 Ω	133.7 Ω
